# Agalsidase alfa in pediatric patients with Fabry disease: a 6.5-year open-label follow-up study

**DOI:** 10.1186/s13023-014-0169-6

**Published:** 2014-11-26

**Authors:** Raphael Schiffmann, Gregory M Pastores, Yeong-Hau H Lien, Victoria Castaneda, Peter Chang, Rick Martin, Anna Wijatyk

**Affiliations:** Institute of Metabolic Disease, Baylor Research Institute, 3812 Elm Street, Dallas, TX 75226 USA; New York University School of Medicine, New York, NY USA; Current affiliation: Department of Medicine/National Centre for Inherited Metabolic Disorders, Mater Misericordiae University Hospital, Dublin, Ireland; Arizona Kidney Disease and Hypertension Center, Tucson, AZ USA; Renown Children’s Hospital, Reno, NV USA; Shire, Lexington, MA USA

**Keywords:** Enzyme replacement therapy, Lysosomal storage disorders, Safety and tolerability, Heart rate variability, Estimated glomerular filtration rate

## Abstract

**Background:**

Signs and symptoms of the X-linked disorder, Fabry disease (FD), can occur early during childhood with heterogeneous clinical manifestations including potential cardiac and renal dysfunction. Several studies support the efficacy of the enzyme replacement therapy (ERT) agalsidase alfa, in adults with FD, though published data on the long-term safety and efficacy of agalsidase alfa in children are limited. As early treatment with ERT has the potential to reduce complications arising from disease progression, children in particular could benefit. The objective of this study was to evaluate the safety and efficacy of long-term agalsidase alfa ERT in children with FD.

**Methods:**

TKT029 was a 6.5-year open-label, multicenter, extension study of children who completed TKT023 (26-week, open-label, every-other-week, intravenous 0.2 mg/kg agalsidase alfa). TKT029 was divided into two phases (before and after an agalsidase alfa manufacturing process change); only patients who participated in both phases were included in the analysis. Primary endpoints included safety, tolerability, and heart rate variability (HRV). Additional efficacy parameters included left ventricular mass index (LVMI), estimated glomerular filtration rate (eGFR), and plasma/urine globotriaosylceramide (Gb_3_).

**Results:**

Eleven patients participated (phase 1 baseline median [range] age: 10.8 [8.6–17.3] years; 10 [90.9%] males). During TKT029 (6.5 years), all patients experienced ≥1 treatment-emergent adverse event (AE); eight patients had ≥1 possibly/probably drug-related AE. Six patients experienced infusion-related AEs, but none discontinued due to AEs. Eight serious AEs arose (two patients); none were deemed drug-related. No deaths occurred. Three patients developed anti-agalsidase alfa antibodies, with IgG antibodies in one patient that were agalsidase alfa neutralizing, but without apparent clinical impact. Renal (eGFR) endpoints remained generally in normal range. Cardiac endpoints remained stable within normal range for LVMI and a trend towards improved HRV, although some patients experienced a reduction in heart rate. Plasma and urinary Gb_3_ reductions were maintained.

**Conclusions:**

TKT029 represents the longest assessment of ERT in children with FD in a clinical trial setting. Overall, agalsidase alfa was well tolerated and demonstrated a stabilizing clinical effect. Agalsidase alfa may be a useful clinical therapeutic option for long-term treatment initiated during childhood in patients with FD.

**Trial registration:**

http://ClinicalTrials.gov identifier NCT00084084.

**Electronic supplementary material:**

The online version of this article (doi:10.1186/s13023-014-0169-6) contains supplementary material, which is available to authorized users.

## Background

The X-linked disorder Fabry disease (FD) is caused by deficient activity of the glycolipid-degrading enzyme α-galactosidase A, which leads to progressive accumulation of the substrate globotriaosylceramide (Gb_3_) in multiple body organs, resulting in potentially severe and ultimately premature fatality [1]. Initial signs and symptoms of FD are manifested during early childhood in both sexes, but especially in affected boys, with symptoms including angiokeratomas, neuropathic pain/acroparesthesia, hypohidrosis, gastrointestinal symptoms, cornea verticillata, and less commonly, cardiac, renal, and cerebrovascular involvement [2].

Enzyme replacement therapy (ERT) is available as a treatment for patients with FD. However, the published literature of ERT safety and efficacy in children with FD is not as robust when compared with adults. Previously, the clinical efficacy and safety of agalsidase alfa was evaluated in 17 children with FD in studies TKT023 and TKT029, conducted over 4 years [3]. Agalsidase alfa was generally well tolerated, and improvements were observed in levels of urine and plasma Gb_3_ concentration, pain severity, and heart rate variability (HRV), while estimated glomerular filtration rate (eGFR) and left ventricular mass indexed to height (LVMI) remained stable.

This study reports on the long-term (6.5 year), open-label, follow-up of patients who qualified for and opted to transition from study TKT023 to an extension trial (TKT029). The objective of extension study TKT029 was to evaluate the safety and efficacy of agalsidase alfa in pediatric patients with FD treated for up to 7 cumulative years.

## Methods

### Study design, patient selection, and treatment

This open-label, multicenter extension study (TKT029; June 10, 2004–June 15, 2011; ClinicalTrials.gov identifier NCT00084084) was designed for pediatric FD patients (7–17 years of age at study enrollment) who had received 6 months of 0.2 mg/kg agalsidase alfa in study TKT023 (August 12, 2002–October 20, 2004) and were within 30 (±7) days of study completion [3]. Together, studies TKT023 (0.5 years) and TKT029 (6.5 years) comprised patients treated for up to 7 years with agalsidase alfa. Study TKT029 was divided into two phases: before (phase 1) and after (phase 2) a change in the agalsidase alfa manufacturing process [3]. Patients were included in this report only if they participated in both phases (transition safety population [TSP]). Phase 2 began ~197 to 223 (mean, 210) weeks after phase 1 baseline agalsidase alfa treatment.

Patients were included if they were determined to be of adequate general health, without potential safety issues or medical contraindications, and had written informed consent provided by a parent or legal guardian. Patients were excluded if they or their legal guardian were deemed unable to understand the study requirements and potential outcomes, or if they were determined by the local investigator as unlikely to follow the study protocol. In both studies TKT023 and TKT029, patients received 0.2 mg/kg body weight of agalsidase alfa every other week, with each intravenous infusion delivered over a 40-minute period.

### Safety and efficacy endpoints

The primary study TKT029 endpoints were safety and tolerability of agalsidase alfa and its effect on HRV. Secondary objectives were to determine the pharmacokinetics of agalsidase alfa at baseline and after treatment initiation, as well as exploratory measurements of renal function (i.e., eGFR), LVMI, and other clinical and patient-reported outcomes (e.g., plasma and urine Gb_3_, pain, health-related quality of life [HRQoL]). As a *post hoc* analysis, urine protein: creatinine ratios were evaluated.

### Safety assessments

Adverse events (AEs) were characterized by severity, potential relationship to study drug and/or infusion, and whether they were classifiable as a serious AE (SAE; an AE that resulted in death, was life-threatening, caused new or prolonged hospitalization, led to persistent disability or congenital abnormality, or was considered an SAE by the treating investigator). Additional safety monitoring included clinical laboratory parameters, vital signs, physical and neurologic examinations, 12-lead electrocardiograms, and potential anti-agalsidase alfa antibody activity evaluated from blood samples screened with an enzyme-linked immunosorbent assay and confirmed by a titration assay. Antibody-positive samples were isotyped (immunoglobulin [Ig] G, IgA, IgM, or IgE) and tested for agalsidase alfa neutralizing activity using an *in vitro* assay [4].

### Efficacy and pharmacodynamic assessments

Cardiac function and structure were assessed through HRV and LVMI, respectively. HRV was assessed via 2-hour Holter monitoring. The time-domain HRV parameters assessed in this study were SDNN (standard deviation [SD] of all filtered RR intervals of the length of the analysis), r-MSSD (square root of the sum of squares of differences between adjacent filtered RR intervals over the length of the analysis), and pNN50 (percentage of differences between adjacent filtered RR intervals that are greater than 50 msec for the whole analysis). SDNN was used as an overall index of HRV, as it encompasses both long- (sympathetic) and short-term (parasympathetic) variability and thus reflects the overall autonomic nervous system activity in the heart. Both r-MSSD and pNN50 describe short-term variability and thus reflect primarily parasympathetic influences on heart rate. A reduction in parasympathetic stimulation of the heart has been reported in male pediatric patients with FD [5]. In addition, heart rate was obtained from 12-lead electrocardiogram measurements. Echocardiogram and the Devereux equations were used to calculate LVMI [6]. LVMI baseline for study TKT029 was week 25/26 of TKT023 (i.e., prior to the first dose of study drug in TKT029, but after the patients had received treatment in TKT023).

Renal function was evaluated through eGFR. The Counahan-Barratt equation was used to calculate eGFR for patients younger than 18 years of age, using height and serum creatinine [7]. For patients who aged to 18 years and over during the study, the Modification of Diet in Renal Disease (MDRD) equation was utilized instead, which factored-in serum creatinine, age, race, and gender [8]. To assess the impact of the equation change at the age of 18 years on the eGFR values, sensitivity analyses were performed using continued eGFR calculated from the Counahan-Barratt equation even after patients turned 18 years old, or the Chronic Kidney Disease Epidemiology Collaboration equation once patients turned 18 years old. As a *post hoc* analysis, 8-hour urine collection was used to estimate urine protein level and proteinuria (defined as urine protein:creatinine ratio ≥0.2).

Plasma and urine Gb_3_ were measured at 8-week intervals in study TKT023 and every 6 months in study TKT029, using a high-performance liquid chromatography assay [9]. Other measurements included the Brief Pain Inventory (severity and interference of pain) [10], Health Utility Index Mark 2 and 3 (HUI; HRQoL) [11,12], and Child Health Questionnaire (CHQ; HRQoL) [13].

### Statistical analysis

The analyses of data are descriptive in nature and no formal inferential statistical tests were performed. For variables following a continuous distribution, tabular summaries consist of descriptive statistics (e.g., means, SDs, medians, minimums, and maximums) and observed and change from baseline values are presented. For categorical and ordinal variables, tabular summaries consist of the number and percentage. For the clinical outcome endpoints (eGFR, plasma and urine sediment Gb_3_, LVMI, and HRV), the percentage change from baseline for these parameters were additionally calculated. As *post hoc* analyses, annualized rate of change (slopes) were estimated for LVMI, eGFR, and urine protein:creatinine ratio based on the random coefficient model using time as a regressor to fit each patient’s data; then, the slopes were estimated by averaging the rate of change across all patients analyzed. This analysis took into account repeated measurements over time and included data from studies TKT023 and TKT029 (phases 1 and 2).

## Results

### Patient disposition and demographic characteristics

**Figure 1:**
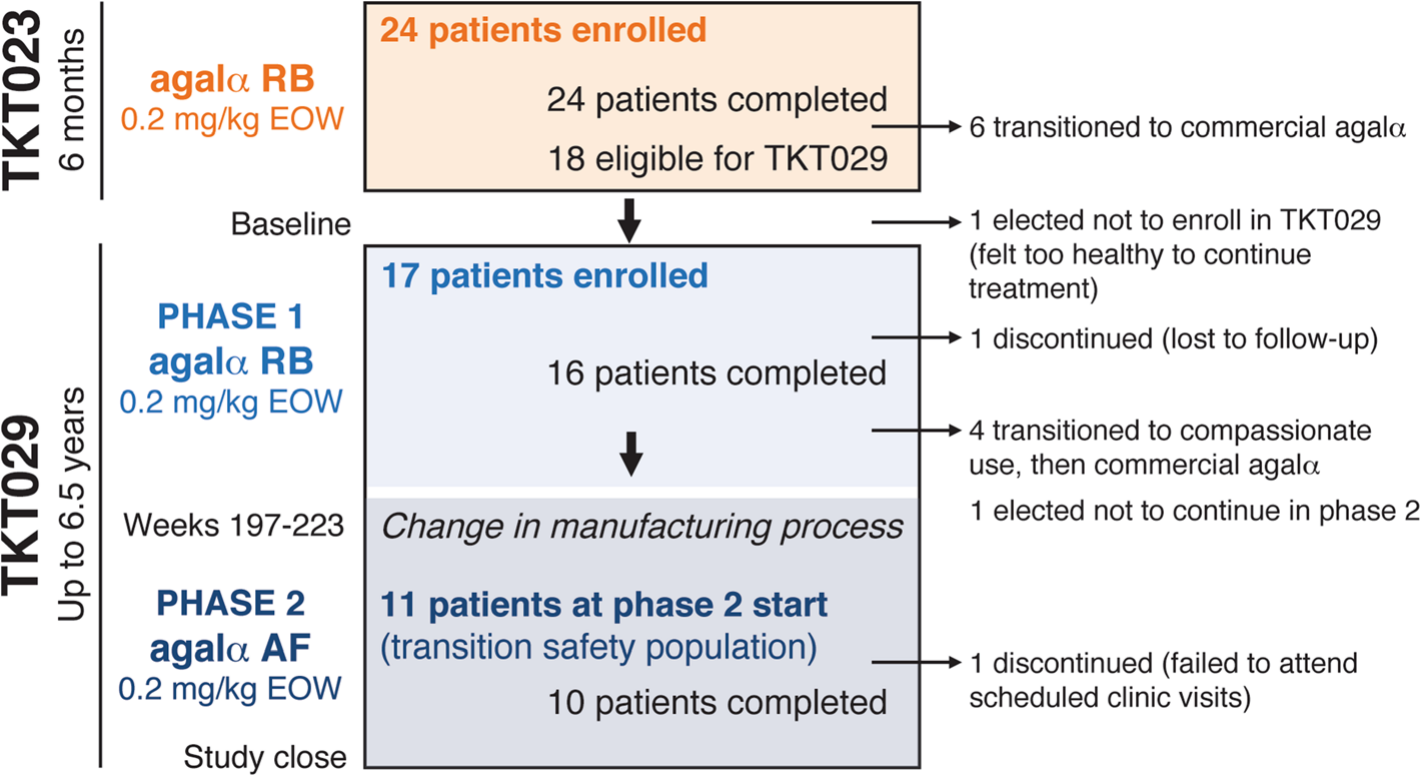
**Timeline and flow of patients in the TKT023 core trial and TKT029 extension study.** Seventeen patients who completed TKT023 enrolled in TKT029. Screening values from TKT023 week 25/26 were used as TKT029 baseline values. TKT029 was divided into two phases, before and after a change in the agalsidase alfa manufacturing process. Patients were transitioned to phase 2 treatment ~197 to 223 (mean, 210) weeks after phase 1 baseline agalsidase alfa treatment. Only patients who participated in both phases (the transition safety population) were analyzed for this report. EOW, every other week; agalα, agalsidase alfa; RB, roller bottle; AF, animal free.

Eleven of 17 patients transitioned from study phase 1 to phase 2 (i.e., the TSP); one patient was lost to follow-up in phase 1, four patients were transitioned first to compassionate-use agalsidase alfa and then to commercial therapy, and one patient elected to not continue with additional treatment (Figure 1). Phase 1 baseline TSP patients’ median (range) age was 10.8 (8.6–17.3) years (10 [90.9%] males, one female [9.1%], nine [81.8%] White). Phase 2 began approximately 4 years after phase 1 baseline; thus, some patients aged past 18 years during the study. One patient failed to attend scheduled clinic visits and was withdrawn from the study. Patients in the TSP had mean ± SD duration of agalsidase alfa treatment of 6.5 ± 0.6 years for a total of 71.9 patient-years of agalsidase alfa exposure.

### Safety profile

The safety profile of 17 patients during phase 1 was described previously [3]. At the end of phase 2, all 11 TSP patients experienced at least one treatment-emergent AE; the majority of these AEs were mild (phase 1: 0; phase 2: 3 [27.3%]) or moderate (phase 1: 5 [45.5%]; phase 2: 6 [54.5%]) in intensity (Table 1). Compared with phase 2, most AEs occurred at a higher incidence in phase 1 wherein the duration of agalsidase alfa exposure was longer. Moreover, fewer patients experienced drug-related, infusion-related, and severe/life-threatening AEs during phase 2. Eight SAEs arose in two patients (patient 1: life-threatening road traffic accident and associated life-threatening traumatic liver injury, facial bones fracture, and renal injury, one mild and one severe cerebrovascular accident, and moderate positional vertigo; patient 2: moderate pectus excavatum requiring surgery), none of which were deemed drug-related. No study discontinuations due to AEs occurred and there were no deaths.

**Table 1:** **Summary of treatment-emergent AEs in the TSP**

**Patients who experienced:**	**TSP,** ***N*** **=11**		
	***n*** **(%)**		
	**Phase 1**	**Phase 2**	**Overall**
≥1 AE	11 (100)	11 (100)	11 (100)
≥1 mild AE	0	3^a^ (27.3)	0^a^
≥1 moderate AE	5 (45.5)	6 (54.5)	5 (45.5)
≥1 severe or life-threatening AE	6 (54.5)	2 (18.2)	6 (54.5)
Most common AEs (≥50% of the TSP overall)			
Cough	9 (81.8)	3 (27.3)	10 (90.9)
Pyrexia	8 (72.7)	3 (27.3)	9 (81.8)
Abdominal pain	7 (63.6)	2 (18.2)	8 (72.7)
Pain in extremity	8 (72.7)	4 (36.4)	8 (72.7)
Chest pain	6 (54.5)	3 (27.3)	7 (63.6)
Headache	7 (63.6)	4 (36.4)	7 (63.6)
Neuralgia	7 (63.6)	0	7 (63.6)
Abdominal pain upper	5 (45.5)	2 (18.2)	6 (54.5)
Diarrhea	3 (27.3)	5 (45.5)	6 (54.5)
Dyspnea	3 (27.3)	3 (27.3)	6 (54.5)
Nasal congestion	6 (54.5)	2 (18.2)	6 (54.5)
Nasopharyngitis	3 (27.3)	4 (36.4)	6 (54.5)
Vomiting	3 (27.3)	3 (27.3)	6 (54.5)
≥1 drug-related AE	8 (72.7)	2 (18.2)	8 (72.7)
≥1 infusion-related AE	5 (45.5)	2 (18.2)	6 (54.5)
≥1 SAE	1 (9.1)	1 (9.1)	2 (18.2)
≥1 drug-related SAE	0	0	0
Discontinuation due to an AE	0	0	0
Death	0	0	0

^a^Highest AE severity was counted at the patient level and the three patients who had “mild” events in phase 2 also had a higher severity event in phase 1 (i.e., moderate, severe, or life-threatening) and are thus classified in a higher severity category in the “Overall” phases (i.e., three mild AEs in phase 2, but none overall).

AE, adverse event; SAE, serious adverse event; TSP, transition study population.

Three patients tested anti-agalsidase antibody positive. One patient was persistently IgG positive with agalsidase alfa neutralizing activity starting at week 55 (of phase 1). Another two patients tested transiently for antibodies (one patient for IgM; one patient for both IgA and IgM), but without neutralizing activity. None of these antibodies had an apparent impact on the incidence of infusion-related AEs. No evidence indicated any potential negative effect based on evaluation of vital signs, neurologic or physical examinations, and clinical laboratory test results.

### Clinical effects of agalsidase alfa

**Figure 2:**
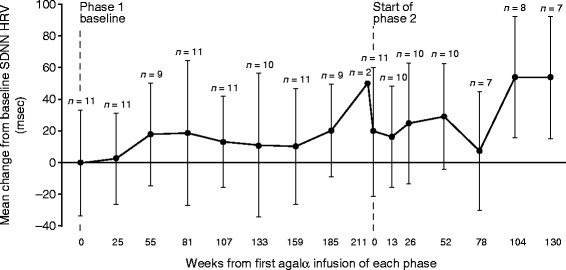
**Change from baseline in heart rate variability (mean ± SD SDNN) by visit in the transition safety population.** HRV assessed by 2-hour Holter monitoring. Baseline mean ± SD SDNN was 91.96 ± 33.21 msec at the beginning of phase 1 (measured at study TKT023 week 25/26). Phase 2 began ~197–223 (mean 210) weeks after phase 1 baseline. Agalα, agalsidase alfa; HRV, heart rate variability; msec, milliseconds; SDNN, standard deviation of all filtered RR intervals for the length of the analysis.

Baseline mean (SD) SDNN was 91.96 (33.21) msec at the beginning of phase 1 (measured at study TKT023 week 25/26). Analysis of SDNN showed that the only negative change from baseline occurred at phase 2 week 13 (mean change of −2.99 msec from phase 2 baseline). All other weeks exhibited consistent mean HRV increases relative to baseline at all time points, with an overall change from phase 1 baseline value of +53.56 msec at the final assessment (Figure 2). Mean SDNN continued an upward trend throughout phase 2 (mean increase of 34.13 msec at the final assessment). Evaluations of r-MSSD and pNN50 showed similar trends for improved HRV over time. At phase 1 baseline, the mean (SD) r-MSSD and pNN50 values were 45.72 (21.57) msec and 16.58% (13.31%), respectively. Mean (SD) r-MSSD increased from 45.72 (21.57) msec (*n* =11) at phase 1 baseline to 59.88 (39.80) msec (*n* =9) at week 185. Mean (SD) pNN50 increased from 16.58% (13.31%) (*n* =11) at phase 1 baseline to 24.11% (22.51%) (*n* =9) at week 185. Insufficient data were available to assess r-MSSD and pNN50 during study phase 2.

**Figure 3:**
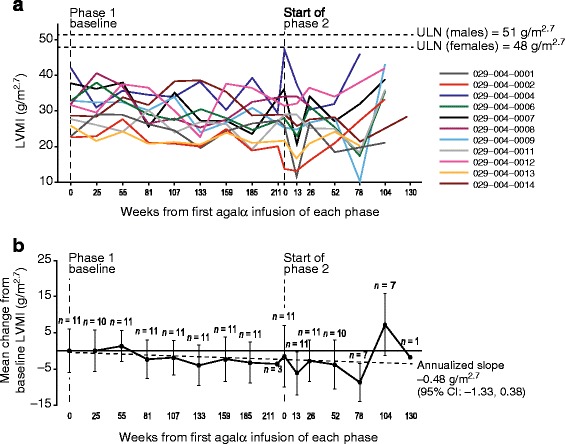
**Left ventricular mass indexed to height (LVMI; g/m** ^**2.7**^ **) over time in the transition safety population. (a)** Individual patients and **(b)** mean (± SD) change from baseline; the dotted line represents the annualized slope. Baseline mean ± SD LVMI values at the beginning of phase 1 were 30.66 ± 5.96 g/m^2.7^ (measured at study TKT023 week 25/26). Agalα, agalsidase alfa; CI, confidence interval; LVMI, left ventricular mass index; SD, standard deviation; ULN, upper limit of normal.

For all patients, baseline LVMI measurements (measured at study TKT023 week 25/26) were within normal range (upper limit of normal range: males =51 g/m^2.7^, females =48 g/m^2.7^), with baseline values ranging from 22.7 to 42.3 (mean ± SD phase 1 baseline 30.66 ± 5.96) g/m^2.7^ (Figure 3). Through phase 1, LVMI decreased from baseline (mean change −3.25 g/m^2.7^ at week 185 in phase 1, which was used instead of the last phase 1 study visit at week 211 that only included three patients) and LVMI continued to decrease through phase 2 (except week 104). Despite LVMI fluctuations observed in individual patients, levels remained below left ventricular hypertrophy criteria throughout the study.

**Figure 4:**
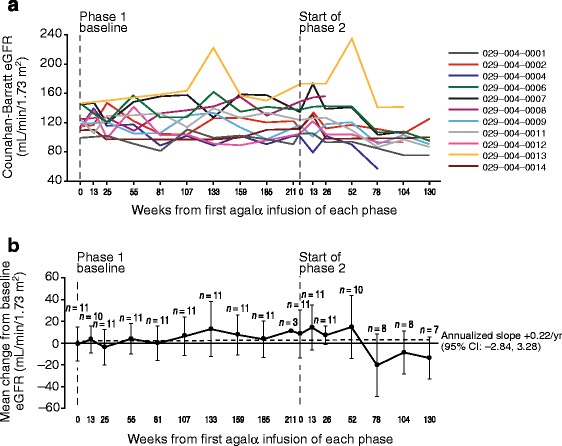
**Estimated glomerular filtration rate (eGFR; mL/min/1.73 m** ^**2**^ **) over time in the transition safety population. (a)** Individual patients and **(b)** mean (± SD) change from baseline; the dotted line represents the annualized slope. Baseline mean ± SD eGFR values at the beginning of phase 1 were 123.29 ± 16.00 mL/min/1.73 m^2^ (measured at study TKT023 week 25/26). Agalα, agalsidase alfa; CI, confidence interval; eGFR, estimated glomerular filtration rate; SD, standard deviation; yr, year.

Mean (SD) phase 1 baseline eGFR was 123.29 (16.00) mL/min/1.73 m^2^. Most individuals showed relatively stable eGFR over time, although a reduction in mean values was observed in the last three phase 2 visits (Figure 4). For some patients, eGFR fluctuated over time, although no consistent trends were detected suggesting clinical deterioration, so these fluctuations were attributed to random variability. One male patient (004–0004; Figure 4a) exhibited eGFR values that declined to 73 mL/min/1.73 m^2^ by end of phase 2. This patient enrolled in study HGT-REP-059 (clinicaltrials.gov identifier NCT01031173) and experienced an eGFR increase during that study for a final level of 110 mL/min/1.73 m^2^, suggesting the eGFR decline was transient. In addition, protein:creatinine ratio (evaluated from available 8-hour urine samples) remained relatively stable over the course of treatment (annualized slope: +0.02 [95% confidence interval (CI): 0.00, 0.03]). Eight of 11 patients entered into proteinuria range (protein:creatinine ratio ≥0.2) during the course of treatment for one (*n* =4), two (*n* =2), or three assessments (*n* =2); none had proteinuria for more than three assessments, nor during their final visit.

**Figure 5:**
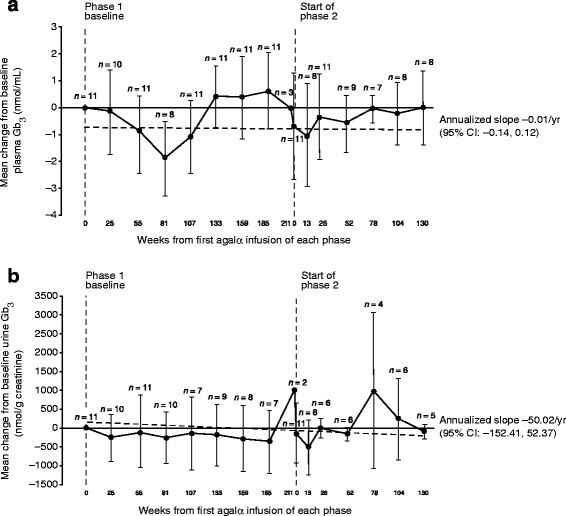
**Mean ± SD change over time in plasma/urine Gb**_**3**_ **in the transition safety population. (a)** Plasma Gb_3_ (nmol/g creatinine) and **(b)** urine Gb_3_ (nmol/mL); the dotted lines represent the annualized slope. Baseline mean ± SD plasma Gb_3_ and urine Gb_3_ values at the beginning of phase 1 were 3.61 ± 1.60 nmol/mL and 538.09 ± 681.27 nmol/g creatinine, respectively (both measured at study TKT023 week 25/26). Agalα, agalsidase alfa; CI, confidence interval; Gb_3_, globotriaosylceramide; SD, standard deviation; yr, year.

Plasma Gb_3_ levels remained below phase 1 baseline (mean ± SD, 3.61 ± 1.60 nmol/mL; measured at study TKT023 week 25/26) for most patients throughout the study (annualized slope −0.01 nmol/mL [95% CI: −0.14, 0.12]; Figure 5a). Reductions in plasma Gb_3_ were maintained in patients with moderately and more severely elevated baseline plasma Gb_3_, except for one patient. In patients with high levels of Gb_3_ at baseline, reductions were seen throughout phases 1 and 2 and, despite transient fluctuations, were maintained at last visit. The observed fluctuations in plasma Gb_3_ were expected based on the biologic variability inherent to this parameter. For patients with normal Gb_3_ levels at baseline, these levels remained normal throughout the study. Similar to the plasma Gb_3_ profile, levels of urine Gb_3_ excretion fluctuated over time, but decreased from the phase 1 baseline value (mean ± SD: 538.09 ± 681.27 nmol/g) with an annualized slope of −50.02 nmol/g (95% CI: −152.41, 52.37; Figure 5b). Urine Gb_3_ excretion remained generally stable, but for some patients, urine Gb_3_ fluctuated over time. However, no trends were found that suggested clinical deterioration.

For other parameters evaluated, no notable changes were detected in any of the HUI items and proxy assessed CHQ scale and summary scores. While a significant reduction in pain severity was seen during TKT023, the reduced pain scores were subsequently maintained and little change was observed in Brief Pain Inventory short form items from baseline to the end of TKT029.

## Discussion

This study represents the longest assessment of ERT for the treatment of children with FD in a clinical trial setting. Agalsidase alfa was generally well tolerated over approximately 7 years of cumulative treatment with most treatment-emergent AEs being mild or moderate in intensity. No treatment-related SAEs were detected; no patients discontinued study participation due to AEs and there were no deaths. The incidence of drug-related and infusion-related AEs decreased over time. No other safety concerns were identified, based on other assessed clinical parameters. Anti-agalsidase alfa antibody formation was low (3/11, 27.3% of the TSP) and with no apparent impact on clinical outcomes. No new safety concerns were identified from all assessed clinical parameters.

HRV improvements originally observed in study TKT023 appeared to be maintained in this extension trial, with an upward trend in SDNN (milliseconds) during phase 2 (SDNN HRV at baseline phase 1: 91.96 msec, by week 130 phase 2: 155.81 msec). A previous study found that normal mean SDNN HRV (by 24-hour Holter monitor) in children increases with age (boys: 57 ± 62 msec [aged 3.4 ± 1.6 years] to 187 ± 38 msec [aged 16.4 ± 0.8 years]; girls: 87 ± 16 msec [aged 2.7 ± 1.8 years] to 201 ± 24 msec [aged 17.4 ± 1.7 years]) [14]. Several patients experienced a reduction in heart rate to below 60 beats per minute based on the 12-lead electrocardiogram, suggestive of bradycardia (Additional file 1: Figure S1). However, bradycardia was not specifically tested for as an endpoint in this study, and no investigators reported an instance of bradycardia as a treatment-emergent AE. Future research would be required to evaluate whether bradycardia is potentially a bradyarrhythmia associated with FD [15,16].

In addition, LVMI remained generally stable and none of the patients reached the adult criteria for left ventricular hypertrophy. Although no studies have been published evaluating annualized slope in LVMI in a population comprising only children with FD, a cross-sectional echocardiographic study of FD in untreated patients (including adults and children) found that males without LVH had a mean (± standard error of the mean) annualized LVMI change of +4.07 ± 1.03 g/m^2.7^ (*n* =39) and females had 2.31 ± 0.81 g/m^2.7^ (*n* =39) [17]. In patients aged <20 years, males experienced a median annualized rate of change of +2.00 g/m^2.7^ (*n* =5) and females changed by +1.36 g/m^2.7^ (*n* =9). In study TKT029 (pediatric, predominantly male patients), LVMI annualized slope was −0.48 (95% CI: −1.33, 0.38) g/m^2.7^. The level of LVMI directly correlates with increasing age [17], so future research would be required for a comparative analysis of current results and the natural history of LVMI in children with FD.

Similarly, renal function was generally stable over the course of the study for most of the patients, albeit with considerable variability during follow-up (e.g., some periods of hyperfiltration with an eGFR >130 mL/min/1.73 m^2^ were observed). Although we are not aware of a study evaluating annualized slope change in eGFR in children with FD, eGFR slopes were assessed in a retrospective chart review study of natural history in a mixed population of adults and children (median [range] age 41.0 years [5.0–77.1]) [18]. In the latter study, males and females (who did not progress to end-stage renal disease) experienced annualized mean eGFR changes of −2.93 mL/min/1.73 m^2^ (*n* =128) and −1.02 mL/min/1.73 m^2^ (*n* =51), respectively. The overall annualized slope in study TKT029 showed a small upward trend of +0.22 (95% CI: −2.84, 3.28) mL/min/1.73 m^2^ through phase 2. As decline in eGFR would be expected to be higher in adult patients compared with children, additional research would be required to determine if early initiation of agalsidase alfa therapy during childhood could have preventive or ameliorating effects on decline in eGFR with aging. The annualized slope of protein:creatinine ratio was relatively stable (+0.02 [95% CI: 0.00, 0.03]), no patients entered the proteinuria range (protein:creatinine ratio ≥0.2) during treatment for more than three assessments, and none had proteinuria during their final visit.

Both plasma and urine Gb_3_ showed decreases from the baseline to the end of study TKT023, and these reductions in Gb_3_ were essentially maintained in study TKT029. The presence of neutralizing anti-agalsidase alfa antibodies has been reported to have a negative effect on the reduction of urine Gb_3_ levels [19]. Here, only one patient developed anti-agalsidase alfa IgG antibodies with neutralizing activity. This patient had a moderately increased level of urine Gb_3_ at baseline that fluctuated above baseline throughout the study and was slightly higher than baseline level at study completion. However, another patient who was anti-agalsidase alfa antibody negative with a moderately elevated level of urine Gb_3_ at baseline, showed fluctuations at various time points, and at study completion had a level similar to that at baseline. It is thus not possible to draw any definitive conclusions regarding the effect of neutralizing anti-agalsidase alfa antibodies on urine Gb_3_ levels in our study.

This study has several limitations. First, TKT029 was not a comparative or placebo-controlled clinical trial, which limits the ability to draw conclusions relative to a control population. In addition, the number of patients in the TSP was small, limiting the power to detect within-patient effects. Because of the lack of a comparator group and low patient number, conducting inferential statistical analyses was impractical, so the results are descriptive only. Furthermore, some patients had transitioned from childhood to adolescence and adulthood. The inclusion of adult patients made evaluation of certain endpoints challenging, as different validated equations needed to be used (e.g., Counahan-Barratt equation for pediatric eGFR versus MDRD for adult eGFR). Nevertheless, we made every effort to conduct sensitivity analyses to verify the results. Urine protein data were assessed from 8-hour urine collection; the accuracy of the estimates may be limited because of inconsistent reporting by individual laboratories as well as missing values. Finally, the prognostic value of HRV in pediatric patients has not been well established, although one study found a reduction in HRV in boys, but not girls [5]. Future studies will be necessary to evaluate proteinuria effects in detail.

## Conclusions

Overall, agalsidase alfa was well tolerated and demonstrated a stabilizing clinical effect. Thus, agalsidase alfa may be a useful clinical therapeutic option for the long-term treatment initiated during childhood in patients with FD.

## Additional file

Additional file 1: Figure S1 Estimated mean (± SD) change from baseline heart rate in the transition safety population. Heart rate (HR) in beats per minute (bpm) was estimated by 12-lead electrocardiography (ECG). Baseline mean ± SD HR at the beginning of phase 1 was 77.8 ± 14.6 bpm (measured at study TKT029 baseline). SD, standard deviation.

## Abbreviations

AE: Adverse event
CI: Confidence interval
eGFR: Estimated glomerular filtration rate
ERT: Enzyme replacement therapy
FD: Fabry Disease
Gb_3_: Globotriaosylceramide
HRQoL: Health-related quality of life
HRV: Heart rate variability
Ig: Immunoglobulin
LVMI: Left ventricular mass index
MDRD: Modification of Diet in Renal Disease
pNN50: Percentage of differences between adjacent filtered RR intervals that are greater than 50 msec for the whole analysis
r-MSSD: Square root of the sum of squares of differences between adjacent filtered RR intervals over the length of the analysis
SD: Standard deviation
SDNN: Standard deviation of all filtered RR intervals for the length of the analysis
SDNN-i/SDNN: Mean of the SDs of all filtered RR intervals for all 5-minute segments of the analysis
SDANN-i/SDANN: SD of the means of all filtered RR intervals for all 5-minute segments of the analysis
TSP: Transition safety population

## References

[1] Brady RO, Gal AE, Bradley RM, Martensson E, Warshaw AL, Laster L (1967). Enzymatic defect in Fabry’s disease. Ceramidetrihexosidase deficiency. N Engl J Med.

[2] Ramaswami U, Parini R, Pintos-Morell G, Mehta A, Beck M, Sunder-Plassmann G (2006). Natural history and effects of enzyme replacement therapy in children and adolescents with Fabry disease. Fabry Disease: Perspectives from 5 Years of FOS.

[3] Schiffmann R, Martin RA, Reimschisel T, Johnson K, Castaneda V, Lien YH, Pastores GM, Kampmann C, Ries M, Clarke JT (2010). Four-year prospective clinical trial of agalsidase alfa in children with Fabry disease. J Pediatr.

[4] Schiffmann R, Kopp JB, Austin HA, Sabnis S, Moore DF, Weibel T, Balow JE, Brady RO (2001). Enzyme replacement therapy in Fabry disease: a randomized controlled trial. JAMA.

[5] Kampmann C, Wiethoff CM, Whybra C, Baehner FA, Mengel E, Beck M (2008). Cardiac manifestations of Anderson-Fabry disease in children and adolescents. Acta Paediatr.

[6] Devereux RB, Alonso DR, Lutas EM, Gottlieb GJ, Campo E, Sachs I, Reichek N (1986). Echocardiographic assessment of left ventricular hypertrophy: comparison to necropsy findings. Am J Cardiol.

[7] Counahan R, Chantler C, Ghazali S, Kirkwood B, Rose F, Barratt TM (1976). Estimation of glomerular filtration rate from plasma creatinine concentration in children. Arch Dis Child.

[8] Levey AS, Coresh J, Greene T, Stevens LA, Zhang YL, Hendriksen S, Kusek JW, Van Lente F (2006). Using standardized serum creatinine values in the modification of diet in renal disease study equation for estimating glomerular filtration rate. Ann Intern Med.

[9] Schiffmann R, Murray GJ, Treco D, Daniel P, Sellos-Moura M, Myers M, Quirk JM, Zirzow GC, Borowski M, Loveday K, Anderson T, Gillespie F, Oliver KL, Jeffries NO, Doo E, Liang TJ, Kreps C, Gunter K, Frei K, Crutchfield K, Selden RF, Brady RO (2000). Infusion of alpha-galactosidase A reduces tissue globotriaosylceramide storage in patients with Fabry disease. Proc Natl Acad Sci U S A.

[10] Cleeland CS, Ryan KM (1994). Pain assessment: global use of the brief pain inventory. Ann Acad Med Singapore.

[11] Feeny D, Furlong W, Torrance GW, Goldsmith CH, Zhu Z, DePauw S, Denton M, Boyle M (2002). Multiattribute and single-attribute utility functions for the health utilities index mark 3 system. Med Care.

[12] Torrance GW, Feeny DH, Furlong WJ, Barr RD, Zhang Y, Wang Q (1996). Multiattribute utility function for a comprehensive health status classification system. Health Utilities Index Mark 2. Med Care.

[13] CHQ: **Child Health Questionnaire**^**™**^**, the HealthActCHQ website**. [https://www.healthactchq.com/chq.php]

[14] Silvetti MS, Drago F, Ragonese P (2001). Heart rate variability in healthy children and adolescents is partially related to age and gender. Int J Cardiol.

[15] Lobo T, Morgan J, Bjorksten A, Nicholls K, Grigg L, Centra E, Becker G (2008). Cardiovascular testing in Fabry disease: exercise capacity reduction, chronotropic incompetence and improved anaerobic threshold after enzyme replacement. Intern Med J.

[16] Shah JS, Hughes DA, Sachdev B, Tome M, Ward D, Lee P, Mehta AB, Elliott PM (2005). Prevalence and clinical significance of cardiac arrhythmia in Anderson-Fabry disease. Am J Cardiol.

[17] Kampmann C, Linhart A, Baehner F, Palecek T, Wiethoff CM, Miebach E, Whybra C, Gal A, Bultas J, Beck M (2008). Onset and progression of the Anderson-Fabry disease related cardiomyopathy. Int J Cardiol.

[18] Schiffmann R, Warnock DG, Banikazemi M, Bultas J, Linthorst GE, Packman S, Sorensen SA, Wilcox WR, Desnick RJ (2009). Fabry disease: progression of nephropathy, and prevalence of cardiac and cerebrovascular events before enzyme replacement therapy. Nephrol Dial Transplant.

[19] Rombach SM, Aerts JM, Poorthuis BJ, Groener JE, Donker-Koopman W, Hendriks E, Mirzaian M, Kuiper S, Wijburg FA, Hollak CE, Linthorst GE: **Long-term effect of antibodies against infused alpha-galactosidase A in Fabry disease on plasma and urinary (lyso) Gb**_**3**_**reduction and treatment outcome.** *PLoS One* 2012, **7:**e47805. 10.1371/journal.pone.0047805 PMC3477102 23094092

