# Supplementary figures and images for: Agalsidase alfa in pediatric patients with Fabry disease: a 6.5-year open-label follow-up study
Source: Orphanet J Rare Dis. 2014 Nov 26;9:169. doi: 10.1186/s13023-014-0169-6 (PMC4260255; doi:10.1186/s13023-014-0169-6)

Mean change from baseline HR by ECG (bpm)

Phase 1  
baseline

Start of  
phase 2

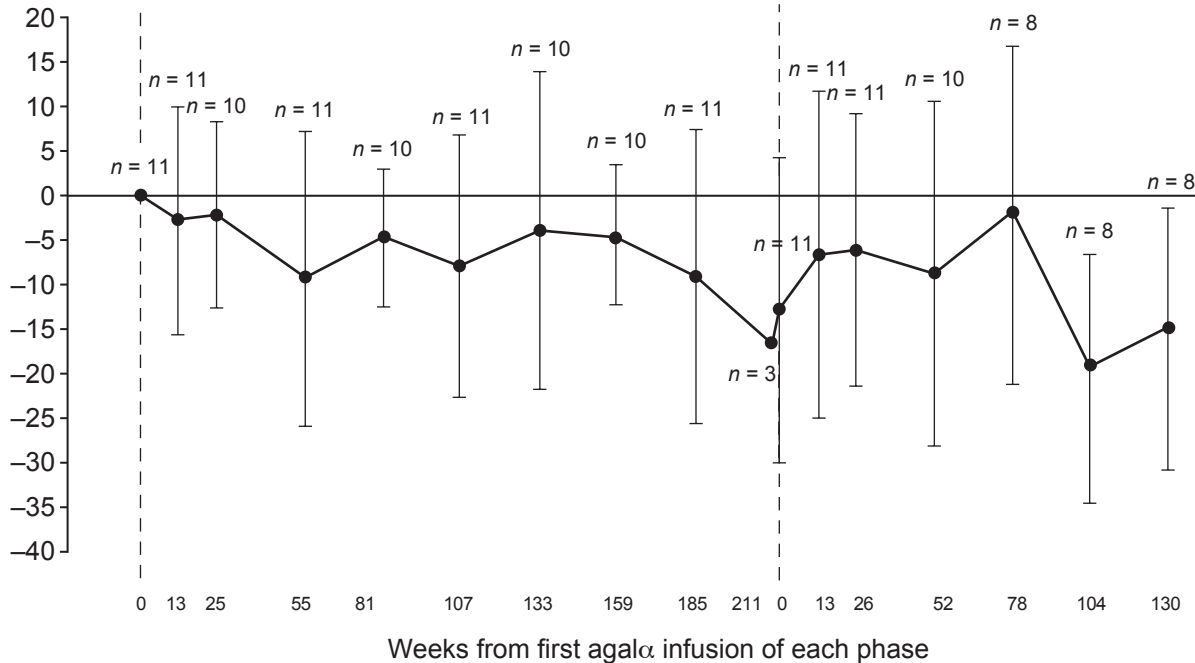

Supplement: Additional file 1: Figure S1 — Estimated mean (± SD) change from baseline heart rate in the transition safety population. Heart rate (HR) in beats per minute (bpm) was estimated by 12-lead electrocardiography (ECG). Baseline mean ± SD HR at the beginning of phase 1 was 77.8 ± 14.6 bpm (measured at study TKT029 baseline). SD, standard deviation. [file 13023_2014_169_MOESM1_ESM.pdf]
